# Dosimetric impact of multileaf collimator leaf width according to sophisticated grade of technique in the IMRT and VMAT planning for pituitary adenoma lesion

**DOI:** 10.18632/oncotarget.12974

**Published:** 2016-10-28

**Authors:** Soo-Min Chae, Ki Woong Lee, Seok Hyun Son

**Affiliations:** ^1^ Department of Radiation Oncology, Cheju Halla General Hospital, Jeju, Korea; ^2^ Department of Radiation Oncology, Incheon St. Mary's Hospital, College of Medicine, The Catholic University of Korea, Seoul, Korea

**Keywords:** multi-leaf collimator, radiosurgery, intensity-modulated radiotherapy, volumetric modulated arc therapy

## Abstract

We analyzed the difference in the dosimetric effect between 5-mm and 2.5-mm multileaf collimator (MLC) leaf width according to the sophisticated grades of intensity-modulated radiotherapy (IMRT) and volumetric-modulated arc therapy (VMAT). Nineteen patients with pituitary adenomas were selected for this study. The treatment plans were performed according to the size of the MLC (5-mm and 2.5-mm MLC), the type of technique (IMRT and VMAT), and the sophisticated grades of each technique (5-field, 9-field, 13-field, 17-field technique in IMRT and 1-arc and 2-arc techniques in VMAT). The downsizing effects of MLC leaf width were analyzed using target volume coverage (TVC), conformity index (CI), dose gradient index (GI), and normal tissue difference 70% isodose line and 50% isodose line. Upon replacing the 5-mm MLC with the 2.5-mm MLC, TVC and CI improved by 1.30% and 1.36%, respectively, in total plans. The TVC and CI improved by 1.68% and 1.67% in IMRT, respectively, and by 0.54% and 0.72% in VMAT, respectively. TVC improved by 2.53%, 1.82%, 1.34%, and 0.94%, and CI also improved by 2.70%, 1.81%, 1.24%, and 0.94%, in 5-field, 9-field, 13-field, and 17-field IMRT, respectively. TVC improved by 0.66% and 0.43%, and CI also improved by 0.93%, and 0.52% in 1-arc and 2-arc VMAT, respectively. Regarding the target coverage, there were dosimetric benefits of a smaller MLC leaf width. However, the downsizing effect of the MLC leaf width decreased with the use of a more precise RT technique and a more sophisticated grade of the same technique.

## INTRODUCTION

Radiotherapy (RT) has been optimized to enable the delivery of a higher dose of radiation to the target volume in order to enhance the treatment results and lower the dose to the surrounding normal tissues to minimize radiation-related complications. Radiosurgery is a leading technique within RT, and includes procedures such as 3-dimensional conformal radiotherapy (3DCRT), dynamic conformal arc therapy (DCAT), and intensity-modulated radiotherapy (IMRT). With development of these planning techniques, the multi-leaf collimator (MLC), which make a shape of the irradiated field to deliver radiation to the target volume, was also developed. The MLC leaf width can be made smaller to create a precise irradiated field acceptable for use in accurate radiosurgery. This micro-MLC has reported to be suitable for radiosurgery in terms of both target volume coverage and normal tissues sparing, regardless of which of the aforementioned techniques is used [1–7].

Generally, IMRT has been considered to be more precise than 3DCRT and DCAT, and the downsizing effect of MLC leaf width is more prominent in 3D-CRT or DCAT than in IMRT [3–6]. Therefore, the sophisticated planning technique may substitute a portion of the downsizing effect of MLC leaf width. In previously reported studies, the downsizing effect of MLC leaf width were compared with respect to the different types of techniques only. Thus, there has been no comparison according to the various sophisticated grades of technique within a particular type of technique [2–6]. Therefore, changing the sophisticated grade within the same technique is necessary in order to evaluate the change of the downsizing effect of MLC leaf width according to the sophisticated grade of technique. In addition, there have been several reports indicating that the downsizing effect of MLC leaf width differs according to the complexity of the target shape [4, 8–10]. Thus, to reduce this kind of bias, relatively simple shapes should be selected for evaluation with respect to the target volume.

In this study, we aimed to verify the downsizing effect of MLC leaf width in both IMRT and volumetric modulated arc therapy (VMAT) in patients with pituitary adenoma, which has a spherical shape, using a 5-mm and 2.5-mm MLC. The downsizing effect of MLC leaf width was analyzed using 4 sophisticated grades of IMRT and 2 sophisticated grades of VMAT within each technique.

## RESULTS

### The downsizing effect of MLC leaf width in overall treatment plans

When using 2.5-mm MLC instead of 5.0-mm MLC, TVC and CI improved by 1.30% ± 0.88% and 1.36% ± 0.98%, respectively. GI improved by 8.67% ± 6.65%, and NTD_70_ and NTD_50_ were 0.92 ± 1.34 cm^3^ and 1.96 ± 2.28 cm^3^, respectively (Table 1).

**Table 1:** Dosimetric improvement ratio according to planning technique and sophisticated grade of each technique

Index	Total	IMRT vs. VMAT			IMRT					VMAT		
		IMRT	VMAT	p value^1^	5-field	9-field	13-field	17-field	p value^2^	1-arc	2-arc	p value^3^
TVC IR (%)	1.30 ± 0.88	1.68 ± 0.84	0.54 ± 0.26	< 0.001	2.53 ± 0.74	1.82 ± 0.68	1.34 ± 0.57	1.02 ± 0.50	< 0.001	0.66 ± 0.27	0.43 ± 0.20	< 0.001
CI IR (%)	1.36 ± 0.98	1.67 ± 1.02	0.72 ± 0.45	< 0.001	2.70 ± 1.10	1.81 ± 0.61	1.24 ± 0.53	0.94 ± 0.48	< 0.001	0.93 ± 0.46	0.52 ± 0.33	< 0.001
GI IR (%)	8.67 ± 6.65	9.10 ± 7.30	7.82 ± 5.11	0.520	9.97 ± 8.10	9.54 ± 9.42	9.00 ± 5.54	7.88 ± 5.85	0.363	7.93 ± 5.46	7.72 ± 4.87	0.936
NTD_70_ (cm^3^)	0.92 ± 1.34	0.99 ± 1.45	0.78 ± 0.96	0.552	0.77 ± 1.47	1.26 ± 2.15	0.93 ± 1.02	1.02 ± 1.18	0.166	0.67 ± 1.07	0.90 ± 0.85	0.530
NTD_50_ (cm^3^)	1.96 ± 2.28	2.11 ± 2.47	1.66 ± 1.81	0.211	1.56 ± 2.32	2.03 ± 2.15	2.35 ± 2.65	2.51 ± 2.91	0.671	1.41 ± 1.85	1.91 ± 1.78	0.990

*Abbreviations*: IMRT = intensity-modulated radiotherapy; VMAT = volumetric-modulated arc therapy; TVC = target volume coverage; CI = conformity index; GI = dose gradient index; IR = improvement ratio; NTD_70_ = normal tissue difference of 70% isodose line; NTD_50_= normal tissue difference of 50% isodose line.

1 independent t-test.

2 paired Friedman test.

3 Wilcoxin signed rank test.

### The downsizing effect of MLC leaf width between IMRT and VMAT

When comparing the downsizing effect of MLC leaf width in IMRT and VMAT, TVC improved by 1.68 ± 0.84% with IMRT and 0.54 ± 0.26% with VMAT (p < 0.001), and CI improved by 1.67 ± 1.02% with IMRT and 0.72 ± 0.45% with VMAT (p < 0.001). Regarding target coverage, the improvement ratio was smaller in VMAT than in IMRT, and the difference was statistically significant.

**Figure 1:**
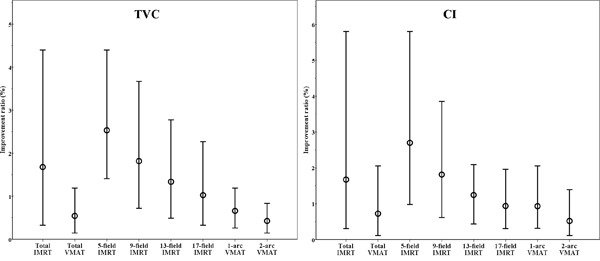
Improvement ratio according to sophisticated grade of each technique for TVC and CI The points presented in this graph indicate the maximum, average, and minimum improvement ratios. Abbreviations: TVC = target volume coverage; CI = conformity index; IMRT = intensity-modulated radiotherapy; VMAT = volumetric-modulated arc therapy.

However, GI improved by 9.10 ± 7.30% with IMRT and 7.82 ± 5.11% with VMAT (p = 0.520). NTD_70_ was 0.99 ± 1.45 cm^3^ with IMRT and 0.78 ± 0.96 cm^3^ with VMAT (p = 0.552), and NTD_50_ was 2.11 ± 2.47 cm^3^ with IMRT and 1.66 ± 1.81 cm^3^ with VMAT (p = 0.211). Regarding normal tissue sparing, there was no statistically significant difference of improvement ratio between IMRT and VMAT (Table 1 and Figure 1).

### The downsizing effect of MLC leaf width according to sophisticated grade of same technique

#### 1. IMRT

By dividing IMRT plans into 5-field, 9-field, 13-field, and 17-field, according to sophisticated grade of treatment technique, TVC improved by 2.53 ± 0.74%, 1.82 ± 0.68%, 1.34 ± 0.57%, and 1.02 ± 0.50% with 5-field, 9-field, 13-field, and 17-field, respectively (p < 0.001). CI improved by 2.70 ± 1.10%, 1.81 ± 0.61, 1.24 ± 0.53%, and 0.94 ± 0.48% with 5-field, 9-field, 13-field, and 17-field, respectively (p < 0.001). With respect to target coverage, there was a statistically significant grade-dependent decrease with the use of a more sophisticated grade of technique from 5-field to 17-field.

With respect to normal tissue sparing, GI improved by 9.97 ± 8.10%, 9.54 ± 9.42%, 9.00 ± 5.54%, and 7.88 ± 5.85%, with 5-field, 9-field, 13-field, and 17-field, respectively (p = 0.363), and there was no statistically significant difference. NTD_70_ were 0.77 ± 1.47 cm^3^, 1.26 ± 2.15 cm^3^, 0.93 ± 1.02 cm^3^, and 1.02 ± 1.18 cm^3^ with 5-field, 9-field, 13-field, and 17-field, respectively (p = 0.166) and NTD_50_ were 1.56 ± 2.32 cm^3^, 2.03 ± 2.15 cm^3^, 2.35 ± 2.65 cm^3^, and 2.51 ± 2.91 cm^3^ with 5-field, 9-field, 13-field, and 17-field, respectively (p = 0.671). There was no statistically significant difference according to the sophisticated grades of this technique (Table 1 and Figure 1).

#### 2. VMAT

By dividing VMAT plans into 1-arc and 2-arc, according to sophisticated grade of treatment technique, TVC improved by 0.66 ± 0.27% and 0.43 ± 0.20% with 1-arc and 2-arc, respectively (p < 0.001), and CI improved by 0.93 ± 0.46% and 0.52 ± 0.33% with 1-arc and 2-arc, respectively (p < 0.001). With respect to target coverage, there was a statistically significant decrease with the use of a more sophisticated grade of technique from 1-arc to 2-arc.

With respect to normal tissue sparing, GI improved by 7.93 ± 5.46% and 7.72 ± 4.87% with 1-arc and 2-arc, respectively (p = 0.936). NTD_70_ was 0.67 ± 1.07 cm^3^ and 0.90 ± 0.85 cm^3^ with 1-arc and 2-arc, respectively (p = 0.530), and NTD_50_ was 1.41 ± 1.85 cm^3^ vs. 1.91 ± 1.78 cm^3^ with 1-arc and 2-arc, respectively (p = 0.990). There was no statistically significant difference according to sophisticated grade of treatment technique (Table 1 and Figure 1).

## DISCUSSION

Radiosurgery is a treatment technique used for delivering highly eradicating radiation dose to the target volume and sparing the surrounding normal tissues [2, 11–13]. Several studies have reported that pa prescribed dose of 20-29 Gy to a pituitary adenoma resulted in an 82-100% tumor control rate, and therefore, in this planning study, we used a prescribed dose of 25 Gy to the target volume [14–18].

MLC, which have undergone continual development in terms of field size and tungsten leaf width over the previous 2 decades, have been classified into standard-MLC with a width greater than 5 mm, and mini-MLC, which has a width below 4 mm. Standard-MLC (5-mm) has been commonly used in conventional RT, whilst 2.5-mm micro-MLC has been used for radiosurgery. As a smaller MLC is able to yield a precise shape for the irradiated field, several studies have reported that downsizing MLC leaf width displayed dosimetric superiority and clinical effectiveness [1-7, 9, 19-22]. Since the development of 4-mm MLC leaf width, several studies have compared the use of 10-mm MLC with that of 4-mm MLC in IMRT [19–22]. Gong et al. reported that, in terms of target coverage, CI and HI improved by 1.1-4.3% and 1.1-1.5%, respectively, and regarding normal tissue sparing, D_5_ of spinal cord, V_10_ and V_20_ of lung, and mean lung dose improved by 4.2%, 2.4%, 3.6% and 2.4%, respectively [21]. Wang et al. reported that, in terms of target coverage, CI and HI improved by 0.9-4.8% and 0.7-0.8%, respectively, whilst with regard to normal tissue sparing, there were no statistically significant difference [22]. Since the size of standard-MLC leaf width was changed from 10 mm to 5 mm, and 2.5-mm micro-MLC has been commonly used in radiosurgery, several studies have reported the effectiveness of 2.5-mm MLC compared with 5-mm MLC [3-5, 7]. Tanyi et al. reported the impact of MLC on 3DCRT, DCAT, and IMRT in 11 cases of liver and 18 cases of lung tumors. With regard to target coverage, CI improved in 62.1%, 55.2% and 51.7% of patients treated with the 3DCRT, DCAT, and IMRT with the use of a 2.5-mm MLC. With respect to normal tissue sparing, volume of normal tissue receiving more than 90% / 50% of the prescription dose was decreased in 72.4% / 86.2%, 69.0% / 79.3%, and 65.5% / 75.9% patients treated with 3D-CRT, DCAT, and IMRT, respectively, in 2.5-mm MLC group [5]. A further study by Tanyi et al. regarding 68 brain radiosurgeries showed that with respect to target coverage, CI improved by 5%, 2.1%, and 1.5% with DCAT, 3DCRT, and IMRT, respectively. With respect to normal tissue sparing, GI improved by 11.7%, 6.4%, and 4.8% with DCAT, 3DCRT, and IMRT, respectively, and peritumoral rind volume receiving 50% of the prescribed dose (PRV50) improved by 18.8%, 12.1%, and 7.2%, % with DCAT, 3DCRT, and IMRT, respectively, in the group who received treatment with 2.5-mm MLC [3]. In this study, TVC and CI improved by 1.30% and 1.36%, respectively when the 2.5-mm MLC was used instead of the 5.0-mm MLC. These results are consistent with the studies previously mentioned above.

The downsizing effect of MLC leaf varied dependent on the treatment technique applied, such as 3DCRT, DCAT, and IMRT, and the effect was most decreased in IMRT, which is more sophisticated technique than 3DCRT and DCAT [3, 5]. In the case of VMAT, single-arc VMAT was similar to multiple static-IMRT; however, multiple-arc VMAT was superior to multiple static-IMRT [23–28]. The downsizing effect of MLC leaf width has predominantly been reported for 3DCRT, DCAT, and IMRT. There have only been a few reports concerning the downsizing effect of MLC leaf width in VMAT [10, 29, 30]. Our previous study regarding the downsizing effect of MLC leaf width in spinal radiosurgery revealed that TVC improved by 8.38% and 2.97% in IMRT and VMAT, respectively, and the effect was smaller with VMAT than with IMRT [10]. In this study comparing IMRT with VMAT, TVC improved by 1.68% and 0.54% with IMRT and VMAT, respectively, and CI improved by 1.67% and 0.72% with IMRT and VMAT, respectively. The downsizing effect of MLC leaf width was found to be smaller in VMAT than IMRT.

In addition, in this study, the downsizing effect of MLC leaf width according to the sophisticated grade of each technique was evaluated. When 2.5-mm MLC was used instead of 5.0-mm MLC, TVC improved by 2.53%, 1.82%, 1.34%, and 0.94% in 5-field, 9-field, 13-field, and 17-field IMRT, respectively, and CI also improved by 2.70%, 1.81%, 1.24%, and 0.94%, in 5-field, 9-field, 13-field, and 17-field IMRT, respectively. These results revealed that the downsizing effect of MLC leaf width decreased with the use of a more sophisticated grade of technique from 5-field to 17-field technique. In the case of VMAT, TVC improved by 0.66% and 0.43% in 1-arc and 2-arc VMAT, respectively, and CI also improved by 0.93%, and 0.52% in 1-arc and 2-arc VMAT, respectively, which revealed that the downsizing effect of MLC leaf width decreased with the use of a more sophisticated grade of technique from 1-arc to 2-arc VMAT. With respect to normal tissue sparing, there were no statistically significant difference according to the sophisticated grade of technique used (IMRT or VMAT). This was because the present study was designed to obtain the maximal target coverage, limiting the dose delivered to 10 Gy for the optic chiasm, and 15 Gy for the brainstem in all plans.

In conclusion, with respect to the target coverage, there were dosimetric benefits of a smaller MLC leaf width, which concurs with previously reported studies. However, the downsizing effect of the MLC leaf width decreased with the use of a more precise RT technique and a more sophisticated grade of the same technique. With the uses of IMRT or VMAT, this effect was very small, with less than 3% improvement ratio, and less than 1% with VMAT. Therefore, further studies are required regarding the necessity for the use of smaller MLC leaf width in the real treatment.

## MATERIALS AND METHODS

### Patient selection

The present study was designed to evaluate the downsizing effect of MLC leaf width according to sophisticated grade of RT technique in IMRT and VMAT by comparing the dose distribution with the use of 5-mm MLC and 2.5-mm MLC. Nineteen patients with pituitary adenomas were selected for this study due to the relatively round shape of the target. Computed tomography (CT) data of all patients were retrospectively collected for this study following institutional review board approval (IRB of Incheon St. Mary's Hospital, the Catholic University of Korea, Reference number: OC16RISI0117).

### Simulation and target delineation

Patients were immobilized using a thermoplastic head mask for the brain prior to simulation. CT scans were performed using the Ingenuity 128-channel CT scanner (Philips Healthcare, Eindhoven, Netherlands) with a 1-mm slice thickness, and Magnetic resonance imaging (MRI) scans were performed with a 1-mm slice thickness. CT and MRI images were imported onto Eclipse version 8.9 (Varian Medical System, Palo, Alto, CA), and image fusion was performed with registration by using Rigid Registration Algorithm version 8.9.17 using pixel data. Target volume and organs at risk (OARs) such as the optic pathway, optic chiasm, and brain stem were contoured based on MRI images. The mean target volume was 6.27 ± 8.83 cm^3^ (range: 0.158 cm^3^ - 31.144 cm^3^).

### Prescription and radiotherapy planning

The prescribed dose was 25 Gy in a single fraction, which was required to treat the functional pituitary adenomas, and a dose of 10 Gy and 8 Gy was allowed to 0.5 cc of the brain stem and 0.2 cc of the optic chiasm/pathway, respectively. In this study, we intended to obtain the maximal target volume coverage whilst satisfying the dose constraints for the brain stem and optic chiasm/pathway. Therefore, the irradiated dose to 0.5 cc of the brain stem and 0.2 cc of the optic chiasm/pathway was almost the same for all treatment plans. However, the coverage rates of the prescribed dose to the target volume varied according to each type of plan. Treatment planning was performed using both 5-mm MLC and 2.5-mm MLC to evaluate the downsizing effect of MLC leaf width. All plans were generated using Eclipse version 8.9 for excluding the bias from different algorithms. For the plan optimization, Dose Volume Optimizer version 8.9 was used for IMRT, and Progressive Resolution Optimizer version 8.9 was used for VMAT. Anisotropic Analytic Algorithm version 8.9 was used for dose calculation. The Fluence map pixel size was 1.5 × 1.5 mm^2.^

IMRT plans consisted of single isocenter, coplanar, and static fields delivered by the sliding-window method (dynamic MLC mode), and the whole gantry angles were 0°, 72°, 144°, 216°, 218° in 5-field, 0°, 40°, 80°, 120°, 160°, 200°, 240°, 280°, 320° in 9-field, 0°, 27°, 54°, 81°, 108°, 135°, 163°, 191°, 219°, 248°, 275°, 302°, 330° in 13-field, and 0°, 21°, 42°, 63°, 84°, 105°, 126°, 147°, 168°, 189°, 210°, 231°, 252°, 273°, 294°, 316°, 338° in 17 field, respectively.

VMAT plans were implemented with a single isocenter, and whole gantry angles were in the counterclockwise direction 179.9-180.1° in 1-arc, and counterclockwise direction 179.9-180.1° plus clockwise direction 180.1-179.9° in 2-arc, respectively.

**Figure 2:**
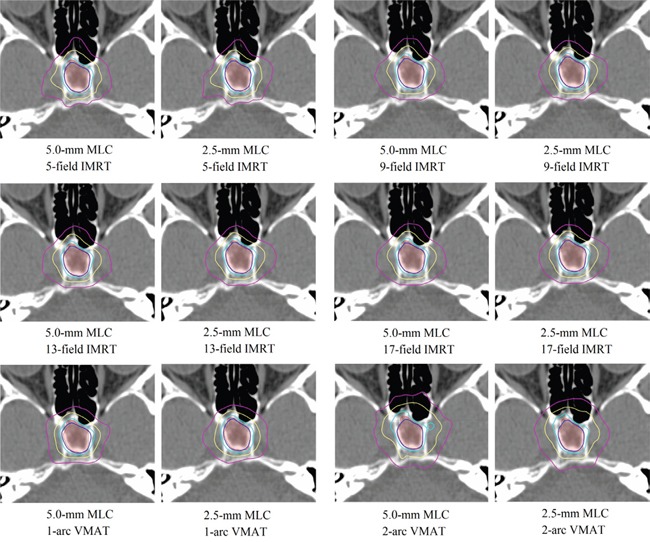
Example of dose distribution in axial plane according to MLC leaf width and sophisticated grades of each technique (the blue line is 100% isodose line, the cyan line is 90% isodose line, yellow line is 70% isodose line, purple line is 50% isodose line) Abbreviations: MLC = multileaf collimator; IMRT = intensity-modulated radiotherapy; VMAT = volumetric-modulated arc therapy.

Finally, 228 treatment plans were generated according to 6 types of sophisticated grades of technique (4 forms of IMRT plans and 2 forms of VMAT plans) and 2 MLC leaf widths for 19 patients. An example of the generated treatment plans is shown in Figure 2.

### Dosimetric indices

To analyze the treatment plan efficiency, we use dosimetric indices, which included Target Volume Coverage (TVC), Conformal Index (CI), Dose Gradient Index (GI), Normal Tissue Difference 70 and 50 (NTD 70 and 50), and improvement ratio (IR).

1. TVC: The index to evaluate the dose coverage to the target volume [31, 32]. The ideal TVC is 100%, and a greater value of TVC indicates a superior dose coverage of the target volume.
  $$\text{TVC}\left(\%\right)=\frac{\text{Volume within the target receiving at least the prescription isodose}}{\text{Target volume}}\times 100\left(\%\right)$$
2. CI: The ratio used to evaluate the quality of fit of the target volume to the prescription isodose volume. The ratio was proposed by the Radiation Therapy Oncology Group (RTOG) and modified by Paddick et al. and Nakamura et al. [11, 33–37]. The ideal CI is 1 and a smaller value of CI indicates a better conformity to the target volume.
  $$\text{Conformityindex}\left(\text{CI}\right)=\frac{\text{PIV }´\text{ TV}}{\text{PT}{\text{V}}_{\text{PIS}}\;´\text{ PT}{\text{V}}_{\text{PIS}}}$$
  [PIV, prescription isodose volume; PTV_PIS_, planning target volume encompassed within the prescription isodose surface; TV, Target volume]
3. GI: The index that represent the degree of dose drop-off outside the target volume, proposed by Paddick et al. [38]. A smaller GI value indicates a better degree of dose drop-off outside the target volume.
  $$\text{Dose gradient index}\left(\text{GI}\right)=\frac{{\text{V}}_{50}}{\text{PT}{\text{V}}_{\text{PIS}}}\;´\;100\left(\%\right)$$
  [V_50_, volume receiving at least 50% of the prescription dose; PTV_PIS_, planning target volume encompassed within the prescription isodose surface]
4. Normal tissue difference 70 & 50 (NTD_70_ & NTD_50_) : The index is defined as the difference between the volume of normal tissue receiving a certain dose utilizing 5-mm MLC, and the volume receiving the same dose using 2.5-mm MLC, in order to assess the degree of normal tissue sparing, and was proposed by Dhabaan et al [7]. The positive greater value of NTD_70_ & NTD_50_ indicates a better sparing of normal tissue
  $$\begin{array}{l} \text{NT}{\text{D}}_{70}=\text{NT}{\text{D}}_{70}^{5-\text{mm MLC}}-\text{NT}{\text{D}}_{70}^{2.5-\text{mm MLC}}\left(\text{c}{\text{m}}^{3}\right)\\ \text{NT}{\text{D}}_{50}=\text{NT}{\text{D}}_{50}^{5-\text{mm MLC}}-\text{NT}{\text{D}}_{50}^{2.5-\text{mm MLC}}\left(\text{c}{\text{m}}^{3}\right) \end{array}$$
  [NTV_70_, Volume of normal tissue receiving 70% of the prescribed isodose; NTV_50_, Volume of normal tissue receiving 50% of the prescribed isodose]
5. Improvement ratio: The ratio used to evaluate the improvement in the index between the two plans (a 2.5-mm MLC plan vs. 5-mm MLC plan) [39].
  $$\text{Improvement ratio}\left(\%\right)=\frac{\left|\text{Inde}{\text{x}}_{2.5-\text{mm MLC}}-\text{Inde}{\text{x}}_{5-\text{mm MLC}}\right|}{\text{Inde}{\text{x}}_{5-\text{mm MLC}}}\times 100\left(\%\right)$$

### Statistical analysis

Statistical analysis was conducted using SPSS for Windows, version 18.0 (SPSS Inc., Chicago, IL, USA) and a p value <0.05 was considered significant. To analyze the effect of MLC leaf width on entire planning and to compare of IMRT and VMAT planning, independent t-test was used. To evaluate the difference in terms of sophisticated grade of IMRT and VMAT, paired Friedman test and Wilcoxon signed rank test were used, respectively.
